# Supplementary material for: Detection of Lymphocytic Choriomeningitis Virus in House Mouse (Mus musculus) in Brazil
Source: Viruses. 2025 Nov 26;17(12):1544. doi: 10.3390/v17121544 (PMC12737706; doi:10.3390/v17121544)
Supplement: Supplementary file 1 [file viruses-17-01544-s001.zip › viruses-3950427-supplementary.pdf]

Table S1. Amino acid and nucleotide similarities of LCMV LBCE strains compared with reference LCMV strains from GenBank.

LBCE Strain	Access number	Strain	Country	Access number	Similarity nt (%)	Strain	Country	Identity aa (%)	Access number
LCMV LBCE20111	PX448801	Libreville_460	GAB	KM882862	87	MS110127	CHN	98	AGP25108
LCMV LBCE20154	PX448802	WE-NIID	JPN	LC413283	86	810366	USA	91	ABA70765
LCMV LBCE20173	PX448803	MX	SVL	Y16308	85	Y	USA	90	AAZ23786
LCMV LBCE20151	PX448804	WE-NIID	JPN	LC413283	86	Y	USA	90	AAZ23786
LCMV LBCE20180	PX448805	WHI	USA	FJ607033	86	MS110127	CHN	99	AGP25108
LCMV LBCE20143	NA	Pasteur	FRA	DQ868485	88	HP65-2009/2	FRA	95	AFI43910
LCMV LBCE20196	PX448806	WE-NIID	JPN	LC413283	88	MS110127	CHN	98	AGP25108
LCMV LBCE20119	PX448807	200501927	USA	FJ607030	87	Y	USA	91	AAZ23786
LCMV LBCE20193	PX448808	MS110127	CHN	KC858138	83	810366	USA	86	ABA70765
LCMV LBCE20188	NA	200501927	USA	FJ607030	80	810366	USA	78	ABA70765
LCMV LBCE20116	PX448809	Makokou	GAB	KM882859	87	MS110127	CHN	98	AGP25108
LCMV LBCE20125	PX448810	WHI	USA	FJ607033	87	MS110127	CHN	98	AGP25108
LCMV LBCE20131	PX448811	JX31	CHN	MG554176	87	Y	USA	91	AAZ23786
LCMV LBCE20150	NA	EEB-7	ESP	JN872495	86	MS110127	CHN	99	AGP25108
